# Correction: Wróbel et al. The GPR39 Receptor Plays an Important Role in the Pathogenesis of Overactive Bladder and Corticosterone-Induced Depression. *Int. J. Mol. Sci.* 2024, *25*, 12630

**DOI:** 10.3390/ijms26178267

**Published:** 2025-08-26

**Authors:** Jan Wróbel, Paulina Iwaniak, Piotr Dobrowolski, Mirosława Chwil, Ilona Sadok, Tomasz Kluz, Artur Wdowiak, Iwona Bojar, Ewa Poleszak, Marcin Misiek, Łukasz Zapała, Ewa M. Urbańska, Andrzej Wróbel

**Affiliations:** 1 Medical Faculty, Medical University of Lublin, 20-093 Lublin, Poland; wrobeljan@onet.eu; 2 Department of Experimental and Clinical Pharmacology, Medical University of Lublin, Jaczewskiego 8b, 20-090 Lublin, Poland; ewa.urbanska@umlub.pl; 3 Department of Functional Anatomy and Cytobiology, Faculty of Biology and Biotechnology, Maria Curie-Sklodowska University, Akademicka 19, 20-033 Lublin, Poland; 4 Department of Botany and Plant Physiology, University of Life Sciences in Lublin, 15 Akademicka St., 20-950 Lublin, Poland; miroslawa.chwil@up.lublin.pl; 5 Department of Chemistry, Institute of Biological Sciences, Faculty of Medicine, Collegium Medicum, The John Paul II Catholic University of Lublin, Konstantynów 1J, 20-708 Lublin, Poland; ilona.sadok@kul.pl; 6 Department of Gynecology, Gynecology Oncology and Obstetrics, Institute of Medical Sciences, Medical College of Rzeszow University, Rejtana 16c, 35-959 Rzeszow, Poland; jtkluz@interia.pl; 7 Obstetrics and Gynecology, Faculty of Health Sciences, Medical University of Lublin, 4-6 Staszica St., 20-081 Lublin, Poland; wdowiakartur@gmail.com; 8 Department of Women’s Health, Institute of Rural Health in Lublin, Jaczewskiego 2 St., 20-090 Lublin, Poland; iwonabojar75@gmail.com; 9 Laboratory of Preclinical Testing, Department of Applied and Social Pharmacy, Medical University of Lublin, 1 Chodźki St., 20-093 Lublin, Poland; ewapoleszak@umlub.pl; 10 Department of Gynecologic Oncology, Holy Cross Cancer Center, 25-377 Kielce, Poland; marcin.misiek@onkol.kielce.pl; 11 Clinic of General, Oncological and Functional Urology, Medical University of Warsaw, Lindleya 4, 02-005 Warsaw, Poland; lzapala@wum.edu.pl; 12 Second Department of Gynecology, Medical University of Lublin, Jaczewskiego 8, 20-090 Lublin, Poland; wrobelandrzej@yahoo.com

In the published manuscript [1], there was an error regarding the affiliation for Marcin Misiek. In addition to affiliation 10, the updated affiliation should include the following: Department of Gynecologic Oncology, Holy Cross Cancer Center, 25-377 Kielce, Poland. The authors state that the scientific conclusions are unaffected. This correction was approved by the Academic Editor. The original publication has also been updated.
